# A Rule-Based Transparent Machine Learning Approach for Precision Crop Protection: Modeling Orchard Microclimatic Orientations and Cherry Fruit Fly Pupal Habitats

**DOI:** 10.3390/insects17090946

**Published:** 2026-09-10

**Authors:** Cebrail Barut, Inanc Ozgen, Bilal Alatas, Halil Bolu, Aytul Yildirim, Ali Murat Tatar

**Affiliations:** 1 Department of Continuing Education Center, Firat University, Elazig 23119, Turkey; cbarut@firat.edu.tr; 2 Department of Bioengineering, Firat University, Elazig 23119, Turkey; iozgen@firat.edu.tr; 3 Department of Software Engineering, Firat University, Elazig 23119, Turkey; balatas@firat.edu.tr; 4 Department of Plant Protection, Dicle University, Diyarbakır 21280, Turkey; 5 Baskil Vocational College, Firat University, Elazig 23800, Turkey; aytulyildirim@firat.edu.tr; 6 Department of Animal Science, Faculty of Agriculture, Dicle University, Diyarbakir 21280, Turkey; tatar@dicle.edu.tr

**Keywords:** explainable artificial intelligence (XAI), cherry fly (*Rhagoletis cerasi*), LightGBM, rule extraction, precision agriculture, edaphic factors

## Abstract

Although traditional machine learning models demonstrate high accuracy in agricultural prediction scenarios, their “black box” nature prevents them from transparently presenting decision-making mechanisms and limits their reliability in integrated pest management (IPM) processes. In this study, the Cherry Fruit Fly (*Rhagoletis cerasi*) was investigated. A rule-based, explainable artificial intelligence (XAI) framework is proposed for characterizing bio-edaphic profiles associated with observed Cherry Fruit Fly Pupal Count (CfPC) density levels and classifying microclimatic aspects (Aspects) using measurable edaphic and biological parameters. The developed hierarchical rule inference engine parses the decision trees of the LightGBM classifier, which achieved the highest performance when benchmarked against 10 baseline machine learning algorithms (11 models in total), and extracts human-interpretable results that can be directly interpreted by experts. In Experiment 1, the analysis characterized the combinations of observed CfPC and edaphic conditions associated with Low, Medium, and High pupal-density profiles, whereas Experiment 2 evaluated the classification of canopy aspect from the measured bio-edaphic variables. According to the derived rules, continuous biological counts (CfPC) serve as the primary biological reference, while edaphic parameters such as soil temperature, pH, lime content, and water saturation percentage characterize additional soil conditions associated with the observed pupal-density profiles. These synthesized rules provide an interpretable representation of the bio-edaphic patterns observed within the studied orchards and may support the development of future precision crop-protection strategies following independent validation.

## Simple Summary

The Cherry Fruit Fly (*Rhagoletis cerasi*) is a major phytophagous pest causing severe yield losses in cherry orchards, yet traditional monitoring relies heavily on broad meteorological data or opaque, “black-box” machine learning models that provide limited insight into the sub-canopy soil conditions associated with the overwintering pupal stage. To address this gap, an explainable artificial intelligence (XAI) framework was developed to parse and extract transparent, human-readable “IF-THEN” rules directly from a LightGBM classifier based on measurable soil parameters (soil temperature, pH, lime content, electrical conductivity, SoilDepth, and saturation percentage) and pupal counts. The proposed LightGBM-based rule extraction engine was benchmarked against 10 baseline machine learning algorithms (11 models in total). In Experiment 1, the framework was used to descriptively characterize bio-edaphic profiles associated with observed CfPC-defined pupal-density categories, whereas Experiment 2 evaluated directional bio-edaphic differentiation beneath tree canopies. By converting complex LightGBM decision tree structures into explicit, human-interpretable threshold rules, the framework provides a transparent representation of the bio-edaphic patterns observed within the studied orchards. These findings may support the future development of precision crop-protection applications, although further independent validation is required before operational field deployment.

## 1. Introduction

Cherries (*Prunus avium* L.), which stand out as a high-value-added product in the context of global fresh fruit trade and agricultural macroeconomics, are commercially cultivated across a wide geographical area, primarily in Türkiye, the United States, Iran, and Italy [1]. Thanks to its ecological advantages, Türkiye leads the world in cherry production and export volume; production areas and orchards are located in Kemalpaşa (Izmir), Manisa, Akşehir (Konya), Sultandağı (Afyon), Uluborlu (Isparta), and Honaz (Denizli), as well as in the Hadim and Taşkent (Konya) regions in recent years, where production areas and orchard stock are expanding rapidly [2]. The “0900 Ziraat” variety is at the heart of these production and export processes. However, sustainable production lines are under threat from biotic stress factors that undermine yield and quality in cherry orchards, particularly in Elazig Province [3]. Among these organisms, the phytophagous species with the most economically devastating impact is known as the Cherry Fruit Fly [*Rhagoletis cerasi* (L.) (Diptera: Tephritidae)]. This pest, which causes fruit drop and rot as a result of the larvae feeding directly on the fruit flesh, can lead to absolute yield losses of up to 100% in the crop if proactive measures are not taken, thereby triggering international quarantine barriers [4]. Although it is known that adults typically emerge from the soil in May and June and begin laying eggs shortly thereafter, the variability of these phenological processes depending on regional microclimatic dynamics is one of the primary focuses of the research ecosystem.

An examination of the pest’s biology revealed that larvae reaching the third instar stage leave the fruit and burrow into the soil beneath the tree’s canopy, where they typically pupate at a depth of 2 to 5 cm [5]. This pupation process, which is completed within the first few hours after the larvae enter the soil, results in the pest spending a significant portion of its life cycle—approximately 10–11 months—in diapause underground. The literature reports that the spatial distribution of pupae is concentrated on the southern and southeastern aspects (orientations) of the tree, and that these areas constitute the highest infection foci [5]. However, uncertainties regarding the microscopic relationships between pupal overwintering success and edaphic parameters remain unresolved. Ecological studies conducted on taxonomically related species, such as the Mediterranean Fruit Fly (*Ceratitis capitata*), have shown that the characteristics of overwintering sites play a deterministic role in adult emergence success [6]. Similarly, it is known that sandy-loam soil structures positively influence pupa development, while clay and silt aggregates suppress adult emergence rates. Furthermore, it has been demonstrated that in species such as Bactrocera dorsalis, soil moisture, shading indices, and particle size directly influence pupation localization. In summary, although it is known that edaphic dynamics shape pupa emergence timing and microclimatic orientation preferences, studies aimed at optimizing these ecological parameters within the framework of integrated pest management (IPM) are quite limited in the literature.

In the field of precision crop protection, artificial intelligence-based approaches for monitoring adult populations have gained significant momentum in recent years. For example, Salamut et al. [7] achieved an average accuracy of 0.9 by comparing deep learning architectures such as Faster R-CNN and RetinaNet for the detection of adult cherry fruit flies on yellow sticky traps. This wave of digitalization focused on object detection continued to evolve in 2026 with the use of the latest YOLO11 architectures for the automatic tracking of closely related species such as Rhagoletis mendax. In addition to image analytics, studies such as that conducted by Karydas et al. [8]—which involved modeling cherry quality using machine learning by fusing UAV-based multispectral data with topographic and end-of-season soil profiles—has validated the potential of multi-source data fusion in precision agriculture. In post-harvest processes, Yazdani et al. [9] successfully classified fruit-level fly egg deformations with accuracy rates exceeding 89% by utilizing hyperspectral imaging algorithms. In terms of making agricultural decision-making processes more transparent, Akyol et al. [10] synthesized human-interpretable logical boundaries by modeling the phenological periods during which cherry fruit fly activity peaks using rule-based evolutionary algorithms (CORE, DMEL, OCEC).

However, these pioneering approaches in the literature have two fundamental shortcomings from both methodological and practical perspectives: First, the overwhelming majority of the deep learning and spectral fusion models developed consist of “black-box” architectures whose internal decision-making mechanisms cannot be deciphered, and thus fail to provide agronomists with the rationale behind their decisions [11]. Second, and more critically, current models focus solely on the adult flight stage or post-harvest physical damage to fruit surfaces; they completely disregard the overwintering mechanisms beneath the trees—which primarily dictate pest population density—as well as real-time edaphic variables (soil temperature, pH, calcium carbonate, and saturation) [12].

To overcome these limitations, there is a growing need in precision agriculture to transition from “black box” machine learning approaches to more transparent models [13]. The application of standard rule learning algorithms allows for the automatic extraction of knowledge in the form of generalized, comprehensible decision rules. This methodology produces models that are directly understandable by human domain experts, such as agriculturists, facilitating proactive decision-making in crop management. By extracting explicit IF-THEN rules from complex agricultural datasets, these systems can accurately diagnose plant states and effectively manage natural resources while remaining highly interpretable [14]. Kaushik and Chatterjee [15] proposed a two-phase framework for automatic ontology construction from domain-specific agricultural texts. In the first phase, they developed the RENT algorithm, utilizing rule-based natural language processing (NLP) to extract single and compound domain terms. In the second phase, a novel rule-based reasoning approach termed RelExOnt was employed to identify both hierarchical and non-hierarchical semantic relationships between the extracted terms. Their findings demonstrated that rule-based NLP techniques achieve high precision in capturing localized terms and complex domain-specific relationships.

XAI in complex modeling generally follows three primary paradigms: post-hoc feature contribution analysis, implicit functional/attention-based interpretability, and deterministic rule extraction. Post-hoc techniques (e.g., SHAP) provide local feature attributions but do not yield exact decision boundaries. Recent hybrid architectures, such as the neural network transformer proposed by Chechkin et al. [16], incorporate specialized functional layers (e.g., Kolmogorov-Arnold Networks) to capture complex, nonlinear feature representations inherently within deep learning frameworks. While such models enhance the internal transparency of neural architectures, they still yield continuous functional representations rather than explicit decision logic. In contrast, deterministic rule extraction converts complex ensemble structures directly into transparent, human-readable “IF-THEN” rules. For agro-ecological and soil-pest modeling, this deterministic approach is crucial, as it provides clear, actionable physical boundaries (e.g., specific pH or temperature thresholds) required for direct field deployment and decision-making.

While conventional pest forecasting systems predominantly rely on macro-meteorological variables (e.g., regional ambient air temperature and precipitation metrics), they frequently fail to account for micro-scale habitat heterogeneity beneath the tree canopy. The overwintering pupal stage of *Rhagoletis cerasi* is directly governed by sub-canopy soil conditions, where local variations in soil temperature, moisture retention, pH, and texture dictate pupal diapause survival and emergence timing. Traditional ecological models often treat soil as a homogeneous substrate, ignoring how microclimatic orientations (aspect) and canopy shading create distinct bio-edaphic niches. To bridge this operational gap in Integrated Pest Management (IPM), this study presents a localized diagnostic framework that integrates fine-scale edaphic properties with biological pupal indicators, offering a site-specific decision-support tool that outperforms broad meteorological approximations.

In this study, to address this multidimensional gap, an innovative and XAI framework is proposed that applies easily measurable soil parameters to a hierarchical rule-based inference architecture. Unlike traditional black-box approaches, the proposed methodology benchmarked the proposed LightGBM model against 10 baseline machine learning algorithms (11 models in total) on our agronomic dataset (*N* = 160) and decomposed the decision tree matrices of the LightGBM classifier—which achieved the highest predictive performance among the evaluated models—at the computational architecture level. In the literature, rule-based algorithms and rule extraction mechanisms are widely used in the development of transparent decision support systems by transforming complex data patterns into human-interpretable deterministic logic frameworks [13,17]. The synthesized Rule Extraction Engine has converted complex gradient weights into deterministic IF-THEN decision rules that provide an interpretable representation of the model-derived bio-edaphic patterns [18,19]. In Experiment 1, the framework was used to characterize the bio-edaphic profiles associated with observed Low, Medium, and High pupal-density levels and to extract interpretable associations between CfPC and the measured edaphic conditions. Accordingly, Experiment 1 is interpreted as a descriptive bio-edaphic profiling and rule-extraction analysis rather than as prospective prediction of unknown pupal density. Similarly, in the microclimatic orchard aspect prediction scenario (Experiment 2), the model demonstrated strong consistency by attaining a macro-average accuracy and weighted F1-score of 95.00%. Overall, the extracted rules provide a transparent representation of the relationships observed between biological pupal abundance and edaphic conditions within the studied orchards and may contribute to the development of future precision crop-protection applications following further validation.

This study incorporates three key innovations aimed at overcoming the methodological, biological, and operational limitations in the existing literature on precision agriculture and digital plant protection:

- Integration of Overwintering Soil Microclimates over Macro-Meteorological and Adult-Focused Approaches: Conventional pest forecasting models rely heavily on macro-meteorological metrics (e.g., regional air temperature, ambient humidity) or focus exclusively on adult flight monitoring and post-harvest fruit damage. In contrast, this study targets the sub-canopy soil environment where *R. cerasi* spends the critical overwintering pupal phase of its life cycle. By mapping fine-scale soil microclimatic parameters (moisture, temperature, pH, lime) directly to pupal survival and distribution, the proposed approach bridges the gap between macro-climatic approximations and site-specific IPM requirements.
- Deterministic Rule Extraction (XAI) for Agronomic Decision-Making: While complex “black box” machine learning models achieve high predictive power, their opaque decision-making limits field applicability for agronomists and farmers. This study translates high-dimensional tree matrices from the LightGBM architecture into deterministic, human-interpretable IF-THEN rules at the feature-split level. This provides explicit and human-interpretable threshold logic with quantified accuracy and support, which can facilitate expert interpretation and subsequent field validation without requiring high computing resources.
- Dual-Focus Bio-Edaphic Modeling of Pest Hotspots and Canopy Microclimates: Using easily measurable edaphic variables (pH, EC, lime content, soil temperature, SoilDepth, and saturation percentage) alongside biological pupal indicators, this study introduces a dual-focal analytical framework. Experiment 1 characterizes the bio-edaphic profiles associated with observed CfPC-defined Low, Medium, and High pupal-density levels and extracts interpretable associations between pupal abundance and the measured edaphic conditions, rather than prospectively predicting unknown pupal density. Experiment 2 explores whether the coupled bio-edaphic patterns discriminate among microclimatic canopy directions (Aspect). To our knowledge, this framework provides an interpretable rule-based characterization of under-canopy micro-habitat patterns through a combined biological and edaphic matrix.

## 2. Materials and Methods

In this study, an innovative methodological framework based on gradient boosted decision trees has been proposed, combining high predictive accuracy with a transparent and interpretable decision support mechanism. In traditional machine learning approaches, complex ensemble models often remain “black boxes,” and the logical processes behind the predictions they produce cannot be directly observed by agricultural experts. To overcome this limitation, a special rule inference engine (Rule Trees) that hierarchically decomposes the tree matrices in the background of the trained powerful LightGBM classifier has been developed. The Extraction Engine has been developed.

To explain how the proposed LightGBM-based rule extraction engine operates at the computational level, the process is executed through four sequential steps:

- Step 1: Disassembling the Tree Structures: Once the LightGBM model is trained, its internal ensemble structure is dumped as a hierarchical JSON/dictionary matrix using the booster_dump_model() interface. This step exposes all individual decision trees and their split nodes (features, operators, and thresholds).
- Step 2: Recursive Path Traversal: A recursive algorithm (collect_rules) traverses each tree from the root node to the leaves. It collects all conditional branches (e.g., pH ≤ 7.55 and Lime ≤ 2.22) that lead to a specific target class prediction.
- Step 3: Calculating Rule Metrics (Support and Accuracy): Every extracted path is evaluated against the training dataset: Sample Support: The number of physical soil samples that fall into the logical boundaries of that specific rule.
  ○ Rule Accuracy: The percentage of those matching samples that actually belong to the predicted target class.
- Step 4: Sifting and Deduplication: To filter out noise and improve field reliability, rules with accuracy below 75% or sample support of fewer than 3 samples are automatically pruned. Duplicate rules generated by different trees are merged, and the remaining rules are ranked according to accuracy. The presented rules represent the highest-accuracy subset selected after this filtering and ranking procedure. Therefore, the accuracy values of 1.00 reported in these tables should not be interpreted as indicating that all retained rules achieved perfect accuracy; retained rules with lower accuracy values also exist. Across the full set of retained rules after applying the predefined accuracy threshold, accuracy values ranged from 80% to 100%, with a mean accuracy of 90%.

**Figure 1:**
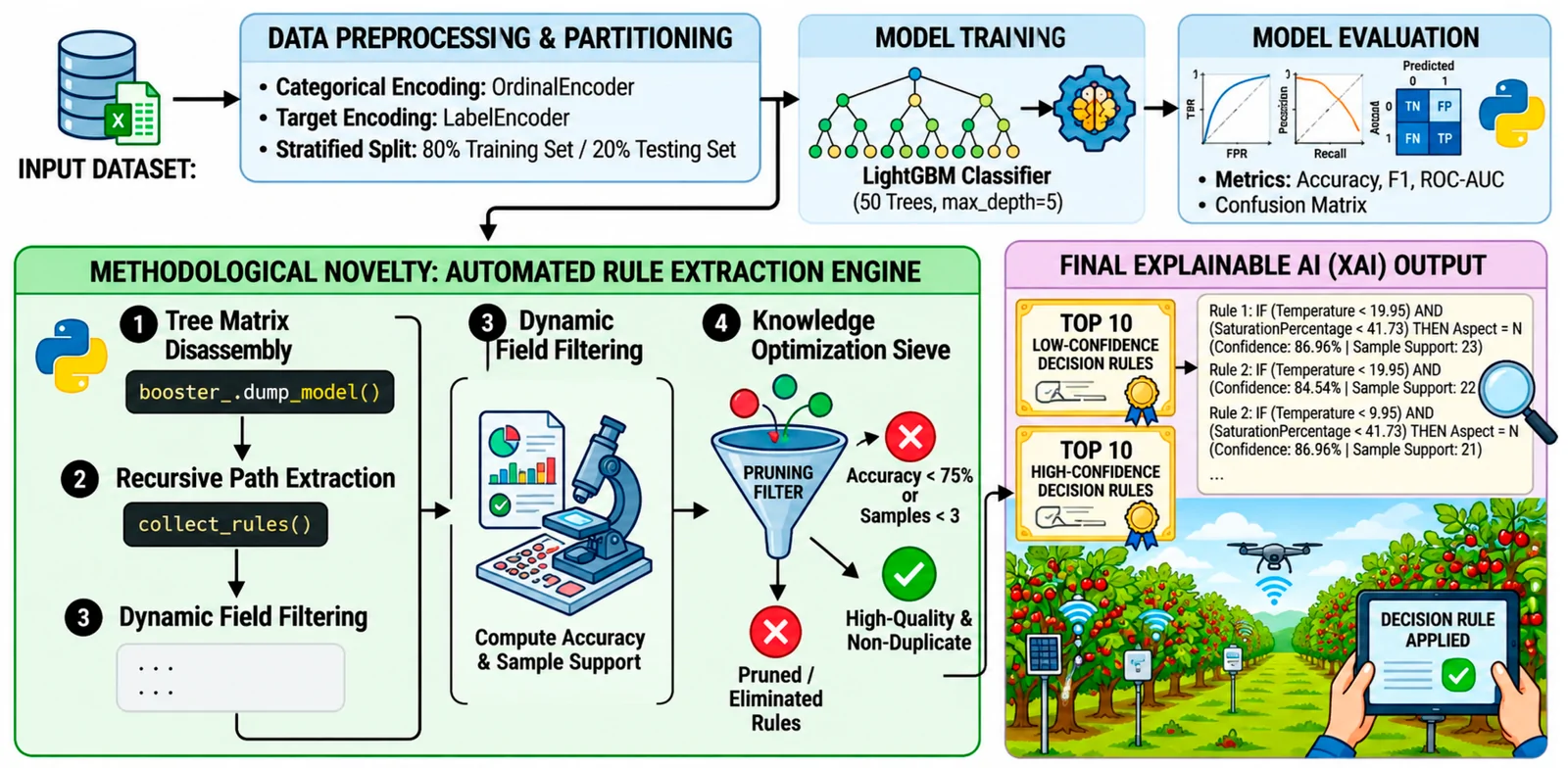
Overall architectural flowchart of the proposed high-accuracy rule extraction engine based on LightGBM tree matrix disassembly for interpretable agricultural data analysis.

Although LightGBM is highly efficient, its leaf-wise tree growth algorithm can be prone to overfitting on moderate-sized datasets (*N* = 160). To rigorously address and mitigate this limitation, we restricted the model’s complexity by enforcing strict regularization constraints during training. Specifically, the maximum tree depth (max_depth) was limited to a range of 3 to 5, the maximum number of leaves (num_leaves) was kept low, and the minimum data points required in a single leaf (min_data_in_leaf) was constrained to prevent the model from isolating outliers. Additionally, our Stratified 5-Fold Cross-Validation design ensured independent validation across folds, while the rule extraction engine’s post-processing pruning (rejecting weak rules with low support or accuracy) effectively eliminated any remaining overfitted patterns. This two-stage validation and filtering pipeline synthesizes the ‘Top 10 High-Accuracy Rules,’ which provide an interpretable representation of the bio-edaphic patterns identified within the present dataset and may support the development of future agricultural management applications (e.g., cherry fruit fly pupal microclimate characterization) following further independent validation. The general architectural flowchart of the proposed method, illustrating the path from data preprocessing and LightGBM training to the dynamic filtering steps of the rule engine and the generation of XAI outputs, is presented in Figure 1.

### 2.1. Experimental Design, Validation Protocol, and Classification Models

Two different independent experimental scenarios were designed to investigate the bidirectional relationship between soil physicochemical properties and pest dynamics:

- Experiment 1—Descriptive Bio-Edaphic Rule Profiling of Pupal Density: Continuous Cherry Fruit Fly Pupal Count (CfPC) was retained together with pH, electrical conductivity (EC), lime content, soil temperature, soil depth, and saturation percentage to characterize combined biological and edaphic profiles associated with the Low, Medium, and High pupal-density categories.

Because the categorical density labels were derived directly from continuous CfPC, Experiment 1 was not interpreted as an independent forward-prediction model for estimating unknown pupal density from soil variables alone. Instead, it was treated as a descriptive bio-edaphic rule-profiling analysis. Accordingly, the high importance of CfPC and the resulting classification performance were not considered evidence of the independent predictive power of the soil variables. The soil variables were evaluated as secondary conditions associated with the observed CfPC-defined density profiles.

- Experiment 2—Exploratory Classification of Canopy Direction: This experiment evaluated whether the North, South, East, and West sectors beneath the tree canopy exhibited distinguishable combinations of soil properties and local pupal abundance. Canopy direction was the response variable, whereas CfPC and the measured soil variables were used as predictors.

The purpose of this experiment was not to develop an operational tool for predicting an already known sampling direction. It was conducted as an exploratory analysis of directional bio-edaphic differentiation beneath the tree canopy.

To objectively evaluate the classification performance and stability of both experimental scenarios, 10 established baseline machine learning algorithms were comparatively benchmarked alongside the proposed LightGBM [20] model (11 models in total) under the same Stratified 5-Fold Cross-Validation framework. These baseline algorithms encompassed ensemble learning methods (Random Forest [21], CatBoost [22], Extra Trees [23]), linear and distance-based models (SVM [24], KNN [25], LDA [26]), rule-based techniques (Decision Tree [27], RIPPER [28], OneR [29]), and artificial neural network architectures (MLP [30]). To prevent data leakage and eliminate evaluation bias, a strict cross-validation protocol was implemented across all experiments. The overall agro-ecological dataset (*N* = 160) was evaluated using Stratified 5-Fold Cross-Validation. In each fold, approximately 80% of the observations were used for model training and the remaining 20% were used for validation, with each observation serving as validation data exactly once across the five folds. The predictions obtained from the validation portion of each fold were subsequently aggregated to generate out-of-fold (OOF) predictions for the complete dataset (*N* = 160). All benchmark model comparisons, including the 10 baseline classifiers and the proposed LightGBM model (11 models in total), were conducted under the same Stratified 5-Fold Cross-Validation scheme using the complete dataset (*N* = 160). In each fold, four class-stratified folds were used for model training and the remaining fold was used for validation. This process was repeated across all five folds so that each observation served as validation data exactly once. The resulting validation predictions were aggregated to generate out-of-fold (OOF) predictions for the complete dataset (*N* = 160). These OOF predictions were used for the reported classification metrics and diagnostic analyses. This cross-validation procedure provides an internal assessment of model performance and stability while reducing evaluation bias; however, it does not constitute independent external validation or establish generalizability to unseen external datasets.

### 2.2. Dataset Description and Soil Analysis

#### 2.2.1. Study Sites and Soil Sampling Design

The study was conducted in two cherry orchards located in Baskil and Harput, Elazig Province, Türkiye. One orchard was selected in each location. Twenty cherry trees were sampled in each orchard, resulting in a total of 40 trees.

Soil samples were collected exclusively from within the canopy projection of the selected cherry trees. For each tree, samples were collected separately from the North, South, East, and West sectors. The SoilDepth variable represents the depth below the soil surface at which pupae were recovered during field sampling. In the dataset, SoilDepth ranged from 0.10 to 0.70 m (10–70 cm). Subsamples obtained within the same directional sector were combined to produce one composite sample of approximately 1 kg. Samples from different canopy directions were not mixed and were treated as separate directional sampling units.

Accordingly, the dataset consisted of 2 orchards × 20 trees × 4 directions = 160 directional soil samples. The location, orchard, canopy direction, sampling depth, and sample code were recorded for each sample.

#### 2.2.2. Recovery and Counting of Pupae

Each approximately 1 kg composite soil sample was transported to the laboratory and processed using a graded sieve system. Sieving was conducted carefully to avoid physically damaging the puparia. Material retained on the sieves was examined under an Olympus SZX7 stereomicroscope (Evident Corporation, Tatsuno, Nagano, Japan), and pupae were manually separated from soil particles, roots, and other organic residues.

Soil particles and organic material adhering to the puparial surface were carefully removed without damaging the specimens. Cleaned pupae were examined under the stereomicroscope only to verify their general condition and to ensure reliable counting. No morphometric measurements, morphological developmental assessments, dissections, diapause evaluations, or controlled adult-emergence experiments were conducted.

The number of eligible pupae recovered from each approximately 1 kg directional composite soil sample was recorded as the Cherry Fruit Fly Pupal Count (CfPC).

#### 2.2.3. Exclusion of Pupae Showing Signs of Suspected Entomopathogenic Infection

After adhering soil particles and organic residues had been removed, the pupae were examined under an Olympus SZX7 stereomicroscope. Pupae showing visible mycelium- or sporulation-like growth, abnormal darkening, softening, collapse, decay, or substantial structural deterioration were considered to show signs of suspected entomopathogenic infection and were excluded from the primary analysis.

Because fungal isolation, culture-based identification, or molecular diagnosis was not performed, the observed external symptoms were not considered definitive evidence of entomopathogenic infection. Therefore, these specimens were described as “pupae showing signs of suspected entomopathogenic infection” rather than as confirmed entomopathogen-infected pupae.

#### 2.2.4. Preparation of Soil Samples

Following pupal separation, the soil samples were air-dried under laboratory conditions, gently disaggregated, and passed through a 2 mm sieve. The prepared samples were subsequently used for physical and chemical soil analyses.

#### 2.2.5. Soil Temperature

Soil temperature was recorded in the field during soil sampling and was associated with the corresponding location, tree, and canopy direction. The recorded temperature value was included in the dataset as the Temperature variable.

#### 2.2.6. Definition of Relative Pupal Density Classes

The number of eligible pupae recovered from each approximately 1 kg directional soil sample was categorized as Low (0–2 pupae), Medium (3–4 pupae), or High (>4 pupae).

The Low, Medium, and High categories were defined by the authors as operational relative-density classes for the present dataset. These classes were not based on previously validated economic injury levels or intervention thresholds. Because *R. cerasi* directly damages marketable cherry fruits, even low pest densities may be biologically relevant. Nevertheless, these categories should not be interpreted as universal economic thresholds until they are validated against adult emergence, trap captures, and fruit infestation rates.

In this study, agro-ecological data records obtained from cherry orchards were analyzed and utilized as inputs for machine learning modeling. This dataset characterizes the physical and chemical state of the soil through five key parameters: pH, electrical conductivity (EC), lime content (% Lime), soil temperature, and saturation percentage. A descriptive statistical summary—including the minimum, maximum, mean, and standard deviation values of these soil parameters—is presented in Table 1. In Experiment 1, CfPC was retained as the primary biological measure of observed pupal abundance. The objective of this analysis was to characterize the bio-edaphic profiles associated with Low, Medium, and High pupal-density levels and to identify interpretable combinations of soil conditions associated with these observed density patterns. Accordingly, the extracted IF–THEN rules were interpreted as descriptive associations between pupal abundance and the measured edaphic variables rather than as prospective predictions of unknown pupal density. To characterize these density profiles with high transparency, the machine-learning and rule-extraction framework was implemented using the algorithmic configurations and validation constraints detailed in Table 2. To prevent overfitting within this low-dimensional feature space (*D* = 7, comprising six edaphic variables and CfPC) on a moderate-sized dataset (*N* = 160), the model complexity was heavily regularized by restricting the maximum tree depth (max_depth) to 5. Furthermore, since the input space is composed of highly specific, five physically grounded edaphic variables together with CfPC as the biological abundance measure, row and feature subsampling rates (bagging_fraction and feature_fraction) were maintained at 1.0, ensuring that all critical edaphic interactions were fully captured during model training. To establish the target response variable (Y) for the classification models, soil samples were categorized into three distinct relative pupal-density classes based on the CfPC:

▪ Low Density (L, *n* = 50): CfPC values of 0–2 pupae per approximately 1 kg directional composite soil sample.
▪ Medium Density (M, *n* = 80): CfPC values of 3–4 pupae per approximately 1 kg directional composite soil sample.
▪ High Density (H, *n* = 30): CfPC values > 4 pupae per approximately 1 kg directional composite soil sample.

In this study, physical and chemical parameters were extracted from two distinct agro-ecological experimental scenarios collected from the under-canopy lines of cherry orchards in Elazig Province to evaluate different biological target outcomes (*N* = 160):

- Experiment 1 (Descriptive Bio-Edaphic Rule Profiling): Continuous CfPC and the measured soil variables were jointly evaluated to characterize the Low, Medium, and High CfPC-defined density profiles. This experiment was interpreted as descriptive rule profiling rather than independent prediction of unknown pupal density from soil variables.
- Experiment 2 (Exploratory Canopy-Direction Classification): Canopy direction was evaluated as the response variable to investigate whether the North, South, East, and West sectors exhibited distinguishable bio-edaphic patterns. This experiment was exploratory and was not intended as an operational tool for predicting an already known sampling direction.

Both experimental setups utilized soil physicochemical parameters (pH, EC, Lime, Temperature, SoilDepth, SaturationPercentage) and Cherry Fruit Fly Pupal Count (CfPC) as independent input predictor features. Model performance and internal performance stability were evaluated using Stratified 5-Fold Cross-Validation by benchmarking 10 baseline machine learning algorithms alongside the proposed LightGBM model (11 models in total).

### 2.3. Model Validation and Experimental Protocol

The dataset consisted of 160 directional soil samples obtained from 40 trees in two cherry orchards. One orchard was located in Baskil and the other in Harput. Four directional samples were obtained from each tree, representing the North, South, East, and West canopy sectors. Model validation was performed using the stratified cross-validation procedure described above. Because samples from the same tree shared tree- and orchard-level conditions, the directional observations were not interpreted as fully independent biological replicates.

## 3. Results

This section presents in detail the machine learning performance of a bidirectional experimental design conducted on an agro-ecological dataset collected from cherry orchards and the findings obtained from the developed transparent rule inference engine. First, the results of Experiment 1, which characterized the bio-edaphic profiles associated with the observed pupal-density classes, are presented, followed by an analysis of Experiment 2, which investigated microclimatic direction discriminability.

### 3.1. Experiment 1: Descriptive Bio-Edaphic Rule-Profiling Results

Experiment 1 was conducted as a descriptive bio-edaphic rule-profiling analysis. Continuous CfPC and the measured soil variables were used jointly to characterize the Low, Medium, and High pupal-density categories.

Because the categorical labels were derived directly from continuous CfPC, the high classification performance and dominant importance of CfPC were mathematically expected. These results should not be interpreted as independent predictions of pupal density from soil variables. The principal purpose of Experiment 1 was to identify the secondary soil conditions accompanying the observed CfPC-defined density profiles.

**Figure 2:**
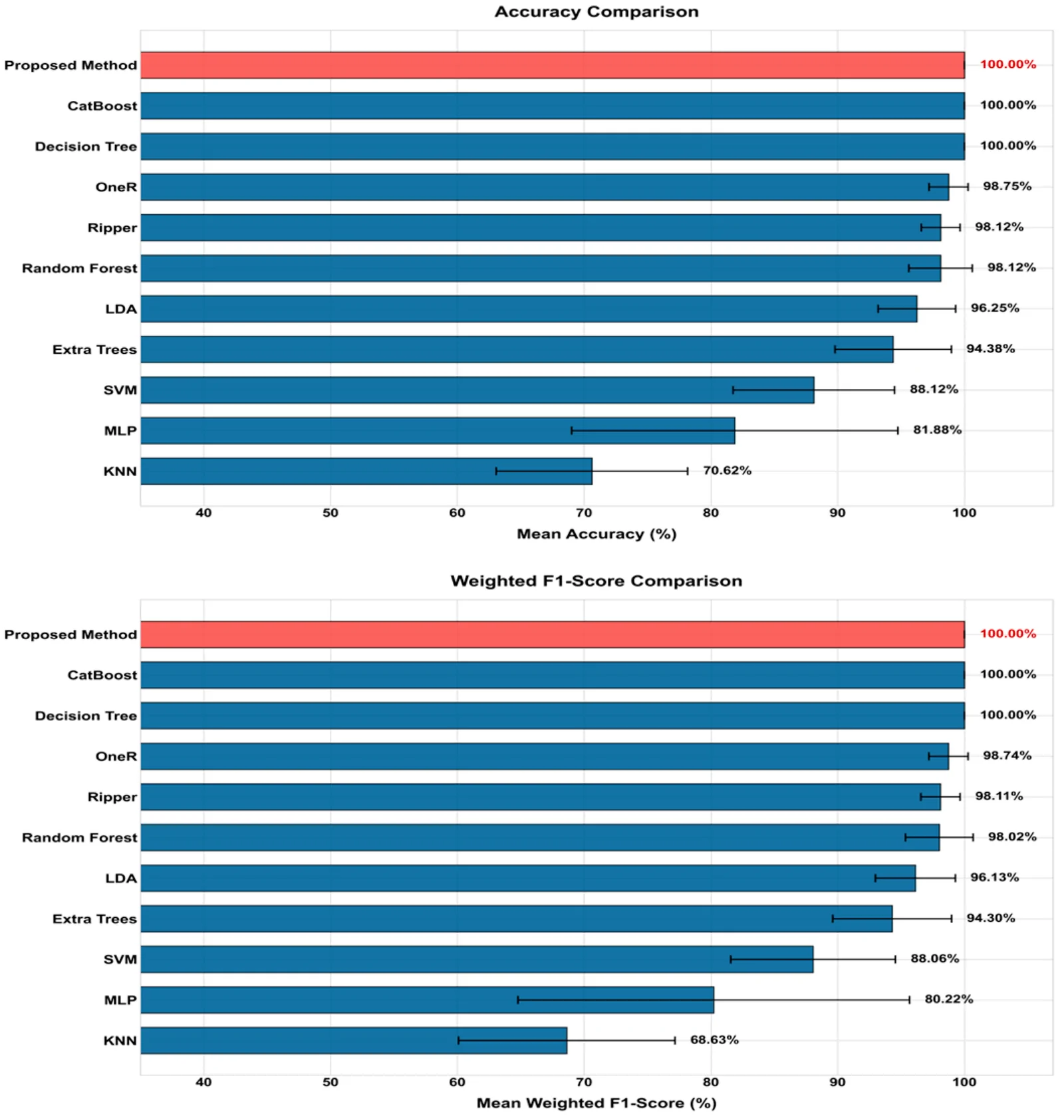
Cross-validated performance benchmark of the proposed LightGBM method and 10 baseline machine learning algorithms (11 models in total) under stratified 5-fold cross-validation. The upper panel displays the Mean Accuracy (%), while the lower panel presents the Mean Weighted F1-Score (%). Black error bars denote the standard deviation across validation folds.

To maintain complete modeling integrity in Experiment 1, the target variable (Y) was strictly defined as the discrete biological pupal concentration classes (Low, Medium, High). The input feature matrix (X) integrates the measured edaphic parameters (soil temperature, pH, electrical conductivity, lime content, and saturation percentage) alongside the biological pupal count (CfPC) to characterize their joint patterns across the CfPC-defined pupal-density classes. The decision rules extracted by the LightGBM rule engine (Table 3) describe non-linear combinations of biological and edaphic conditions associated with the observed under-canopy pupal-density profiles. Accordingly, these rules are interpreted as descriptive bio-edaphic associations rather than as prospective soil-based predictions of unknown pupal population levels. The overall results obtained by benchmarking the proposed LightGBM model against 10 baseline machine learning algorithms (11 models in total) under the same Stratified 5-Fold Cross-Validation framework and consistent randomness settings are presented in Figure 2.

The comparative performance of all 11 models, comprising the proposed LightGBM method and 10 baseline classifiers, evaluated under a stratified 5-fold cross-validation scheme is illustrated in Figure 2. The proposed method achieved a Mean Accuracy of 100.00% and a Mean Weighted F1-Score of 100.00%. CatBoost and Decision Tree also achieved 100.00% Mean Accuracy and 100.00% Mean Weighted F1-Score. OneR achieved 98.75% Mean Accuracy and 98.74% Mean Weighted F1-Score, followed by RIPPER (98.12% accuracy/98.11% weighted F1-score) and Random Forest (98.12% accuracy/98.02% weighted F1-score). LDA achieved 96.25% Mean Accuracy and a 96.13% Mean Weighted F1-Score, while Extra Trees achieved 94.38% accuracy and a 94.30% weighted F1-score. SVM achieved 88.12% Mean Accuracy and an 88.06% Mean Weighted F1-Score. MLP showed lower performance (81.88% accuracy/80.22% weighted F1-score), while KNN yielded the lowest overall metrics (70.62% accuracy/68.63% weighted F1-score). The error bars in Figure 2 represent the standard deviation across the five cross-validation folds and indicate the degree of internal performance variability for each classifier.

The decision rules summarized in Table 3 describe combinations of observed CfPC and soil physicochemical conditions within the present dataset. These rules represent site-specific statistical associations and should not be interpreted as experimentally demonstrated physiological tolerance limits or independent soil-based predictions of pupal density.

While the strong classification performance across tree-based algorithms highlights clear deterministic signals between soil physicochemical thresholds and pupal density classes, these findings must be interpreted within the structural boundary of the dataset. Because the sampling was conducted within a single geographic region and single agricultural season (*N* = 160), environmental background noise was naturally minimized. The applied LightGBM-based rule pruning, combined with Stratified 5-Fold Cross-Validation, was used to reduce the risk of overfitting and to assess internal model stability within the present dataset. However, the lack of an independent external validation dataset from geographically and microclimatically distinct orchards remains a primary limitation of this study. Future work will focus on testing these extracted rule sets across multi-seasonal and regional datasets to assess the true boundaries of model generalization.

The performance outputs presented in Table 4 summarize the class-specific out-of-fold performance of the proposed LightGBM framework under Stratified 5-Fold Cross-Validation for the CfPC-defined pupal-density classes (H, L, M). Across the aggregated out-of-fold predictions (*N* = 160), the model achieved an overall classification accuracy of 1.0000 (100.00%). Class-specific analysis showed a Precision, Recall, and F1-score of 1.0000 for the High-density (*H*; *n* = 30), Low-density (*L*; *n* = 50), and Medium-density (*M*; *n* = 80) classes. Consequently, both the Macro Average and Weighted Average Precision, Recall, and F1-score values were 1.0000.

The perfect separation observed in this internal cross-validation analysis should, however, be interpreted in the context of the design of Experiment 1. Because the Low, Medium, and High density classes were deterministically derived from continuous CfPC and continuous CfPC was retained among the input variables, the strong discrimination of the CfPC-defined classes was expected. Therefore, these performance values are not interpreted as evidence that the measured edaphic variables alone can prospectively predict unknown pupal density. Rather, Experiment 1 uses the LightGBM framework to characterize the observed CfPC-defined density profiles and to extract interpretable bio-edaphic associations accompanying these profiles.

**Figure 3:**
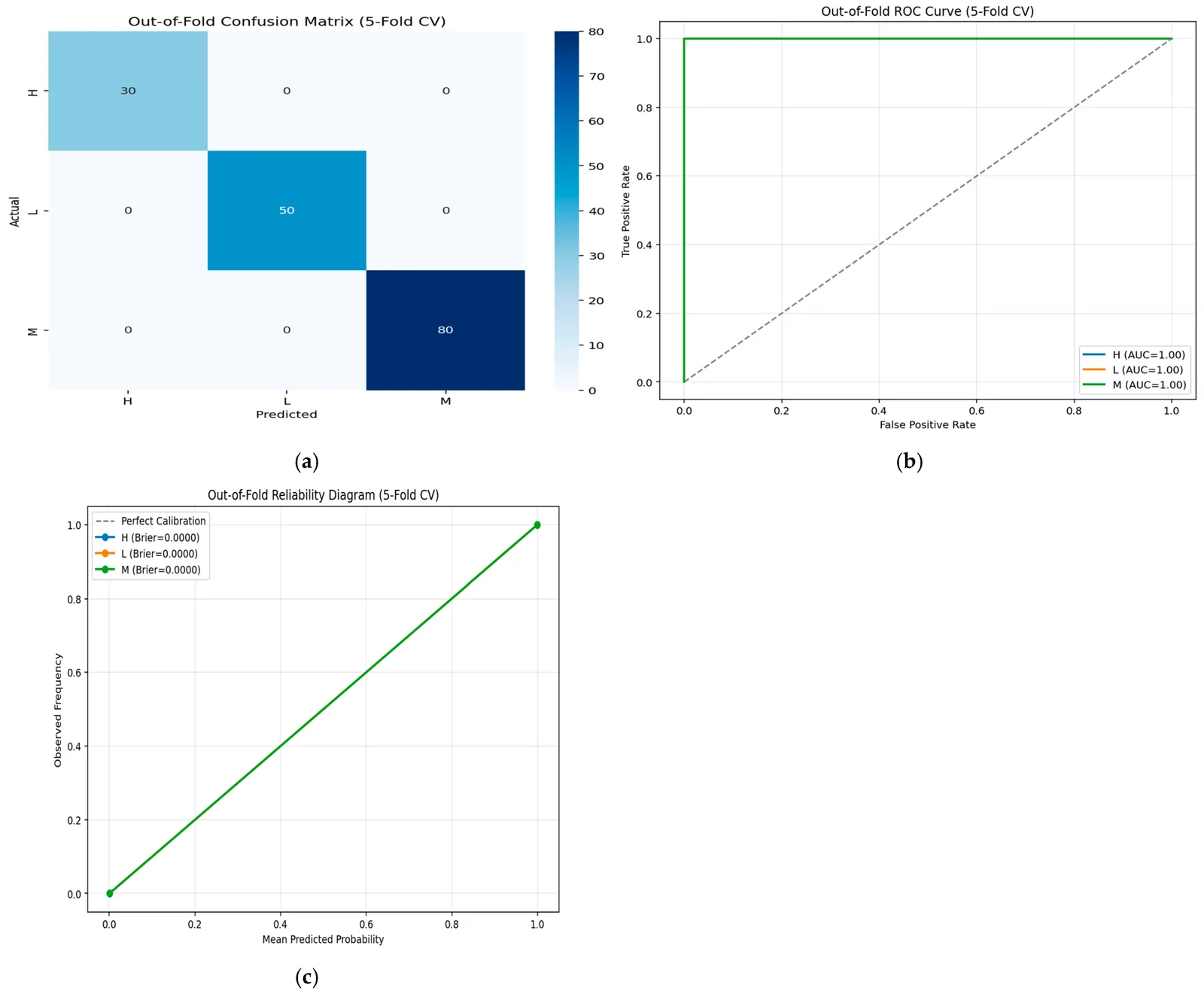
Statistical diagnostic panel for the proposed LightGBM framework for CfPC-defined pupal-density classification based on aggregated out-of-fold predictions generated through Stratified 5-Fold Cross-Validation: (**a**) confusion matrix displaying classification counts across the three classes (H, L, M); (**b**) multi-class Receiver Operating Characteristic (ROC) curves with class-wise Area Under the Curve (AUC) values, where the dotted diagonal line represents the no-discrimination (random classifier) reference; and (**c**) reliability diagram and class-wise Brier scores assessing probability calibration.

To address statistical validity and model reliability, the performance of the proposed LightGBM framework was evaluated using out-of-fold predictions generated through stratified 5-fold cross-validation across the complete dataset (*N* = 160), as presented in Figure 3.

- Confusion Matrix Analysis (Figure 3a): The out-of-fold confusion matrix obtained from the stratified 5-fold cross-validation procedure showed an overall classification accuracy of 100.00% across all 160 observations. As shown in Figure 3a, the model correctly classified all 30 observations of Class H, all 50 observations of Class L, and all 80 observations of Class M, with no misclassifications between any of the three classes. Because each observation was predicted only when it belonged to the corresponding validation fold, these results represent aggregated out-of-fold classification performance rather than training-set performance.
- Multi-Class ROC Analysis (Figure 3b): Multi-class Receiver Operating Characteristic (ROC) analysis was performed using the out-of-fold class-probability estimates obtained across the five validation folds. The model achieved an AUC of 1.00 for each of the three classes (H, L, and M), indicating complete discrimination among the CfPC-defined density classes within the present cross-validation setting.
- Model Calibration Assessment (Figure 3c): The reliability diagram was constructed from the out-of-fold predicted probabilities rather than in-sample predictions. The resulting Brier scores were 0.0000 for Class H, 0.0000 for Class L, and 0.0000 for Class M, with the reliability curves coinciding with the ideal calibration reference. These results indicate perfect probability agreement within the present out-of-fold evaluation. However, given that the density classes in Experiment 1 are deterministically derived from CfPC and continuous CfPC is retained among the input variables, this perfect internal performance should not be interpreted as evidence of prospective soil-only prediction, external generalizability, or independent model calibration.

### 3.2. Experiment 2: Aspect Estimation Results and Discussion

**Figure 4:**
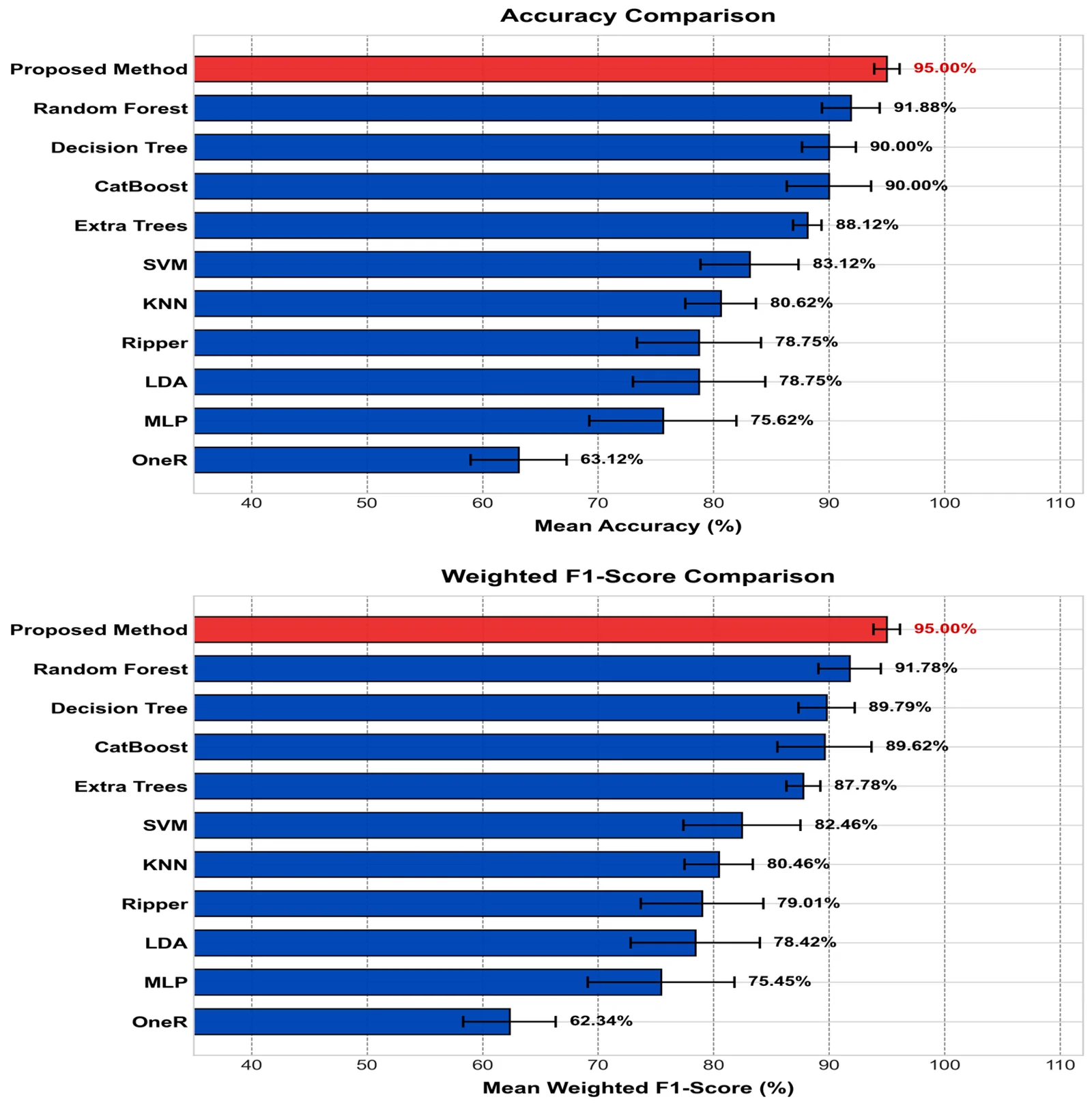
Cross-validated performance benchmark of 10 baseline machine learning algorithms and the proposed LightGBM model (11 models in total) for canopy aspect classification (Experiment 2) under Stratified 5-Fold Cross-Validation. The upper panel presents the Mean Accuracy (%), while the lower panel presents the Mean Weighted F1-Score (%). Error bars represent the standard deviation across the five validation folds.

In Experiment 2, the prediction scenario evaluated the microclimatic Aspect orientation classes (North, South, East, West) as the target variable (Y). In this setup, local biological pupal density (CfPC) served as an independent ecological predictor (X) alongside the five edaphic soil properties (pH, EC, Lime, Temperature, SoilDepth, and SaturationPercentage). This formulation allows the rule engine (Table 5) to capture how pest pupation density interacts with soil thermal and moisture regimes across different canopy orientations due to variable solar exposure. Directional (north, south, east, west) microclimatic differences below the tree canopy directly affect soil physicochemical characteristics due to ecological factors such as sunshine duration, moisture-holding capacity, and wind exposure. In this experimental scenario, the performance of soil parameters taken from cherry orchards in distinguishing the aspect from which the sample was taken was tested. Cross-validated performance metrics of the proposed LightGBM model and 10 baseline machine learning algorithms (11 models in total) evaluated in this study are presented in Figure 4.

The cross-validated classification performance of the 10 baseline algorithms and the proposed LightGBM model (11 models in total) for canopy directional aspect classification (Experiment 2) is presented in Figure 4. The reported results represent internal performance estimates obtained under the Stratified 5-Fold Cross-Validation framework. Functioning as the primary leakage-free predictive benchmark of this study, the proposed method achieved a top-tier Mean Accuracy of 95.00% and a Mean Weighted F1-Score of 95.00%. Strong competitive results were recorded by Random Forest (91.88% accuracy/91.78% F1-score), while standard Decision Trees and CatBoost achieved identical mean accuracies of 90.00%, with weighted F1-scores of 89.79% and 89.62%, respectively. Mid-range classifiers demonstrated moderate predictive capacity, led by Extra Trees (88.12% accuracy/87.78% F1-score), SVM (83.12% accuracy/82.46% F1-score), and KNN (80.62% accuracy/80.46% F1-score). These were followed by rule-based Ripper (78.75% accuracy/79.01% F1-score) and Linear Discriminant Analysis (LDA: 78.75% accuracy/78.42% F1-score). Multi-Layer Perceptron (MLP) achieved a 75.62% accuracy and 75.45% F1-score, whereas the single-rule baseline OneR yielded the lowest overall metrics (63.12% accuracy/62.34% F1-score). Notably, the relatively narrow error bars in Figure 4 indicate low performance variability across the five validation folds, suggesting stable internal cross-validation performance of the proposed approach in classifying directional microclimatic orientations using biological and soil covariates. The LightGBM model, which demonstrated 95% aggregated out-of-fold accuracy in the Aspect prediction scenario, was analyzed using the proposed XAI rule filtering engine. Following the pruning steps, the model was filtered through hierarchical decision mechanisms and exhibited directional behavior. Some well-defined rules for deciphering microclimate-soil interaction are summarized in Table 5.

The rule patterns obtained in Table 5 are directional below the tree crown line. This clearly confirms the physical traces left by orientation on soil parameters. The microclimatic analysis of the rules highlights the following key points:

- North (N) Aspect: Characterized by lower soil temperatures (10.20–11.65 °C) and narrower water saturation ranges (38.10–41.70%) due to reduced direct solar exposure and shaded conditions.
- South (S) Aspect: Characterized by higher saturation tolerances (up to 49.00%) and moderate temperature profiles reflecting increased daily solar radiation.
- West (W) Aspect: Exhibits the highest thermal accumulation, with soil temperatures reaching up to 12.70 °C. Notably, pupal counts remain low (CfPC ≤ 1.50) under these conditions, highlighting the suppressive impact of elevated temperatures and evaporation on pupal survival.
- East (E) Aspect: Distinctive edaphic boundaries become particularly pronounced within shallow soil layers (0.10–0.17 m depth), accompanied by lower lime contents (1.62–1.82%)

These synthesized, explainable rules reflect how directional microclimates modulate the soil environment under the tree canopy. Understanding these directional dynamics within edaphic layers provides agricultural experts with practical, directly applicable insights for targeted pest management.

Beyond the overall classification performance of the algorithms, classification report metrics were examined to assess class-specific performance for the microclimatic orientation classes (E, N, S, W). The class-based Precision, Recall, and F1-Score values of the proposed LightGBM framework are presented in Table 6. The performance outputs presented in Table 6 summarize the class-specific results obtained from out-of-fold predictions generated through Stratified 5-Fold Cross-Validation for the microclimatic Aspect estimation scenario. Across the combined out-of-fold predictions (*N* = 160), the model achieved an overall classification accuracy of 0.9500 (95.00%), with a macro-average F1-score of 0.9497 and a weighted-average F1-score of 0.9497.

Class-specific results showed consistently high performance across the four orientation categories. The East (E) aspect achieved a Precision of 0.9091, a Recall of 1.0000, and an F1-score of 0.9524, indicating that all 40 East observations were correctly identified, although some observations from other classes were predicted as East. The North (N) aspect achieved a Precision of 1.0000, a Recall of 0.8750, and an F1-score of 0.9333. The South (S) aspect achieved a Precision of 0.9744, a Recall of 0.9500, and an F1-score of 0.9620, while the West (W) aspect achieved a Precision of 0.9286, a Recall of 0.9750, and an F1-score of 0.9512.

With a balanced sample distribution across the four classes (*n* = 40 per aspect class), the macro-average and weighted-average metrics were identical: Precision = 0.9530, Recall = 0.9500, and F1-score = 0.9497. This agreement is expected because each class contributes equally to the evaluation. Overall, the aggregated out-of-fold results indicate consistently high class-specific discrimination within the present dataset under the Stratified 5-Fold Cross-Validation procedure.

**Figure 5:**
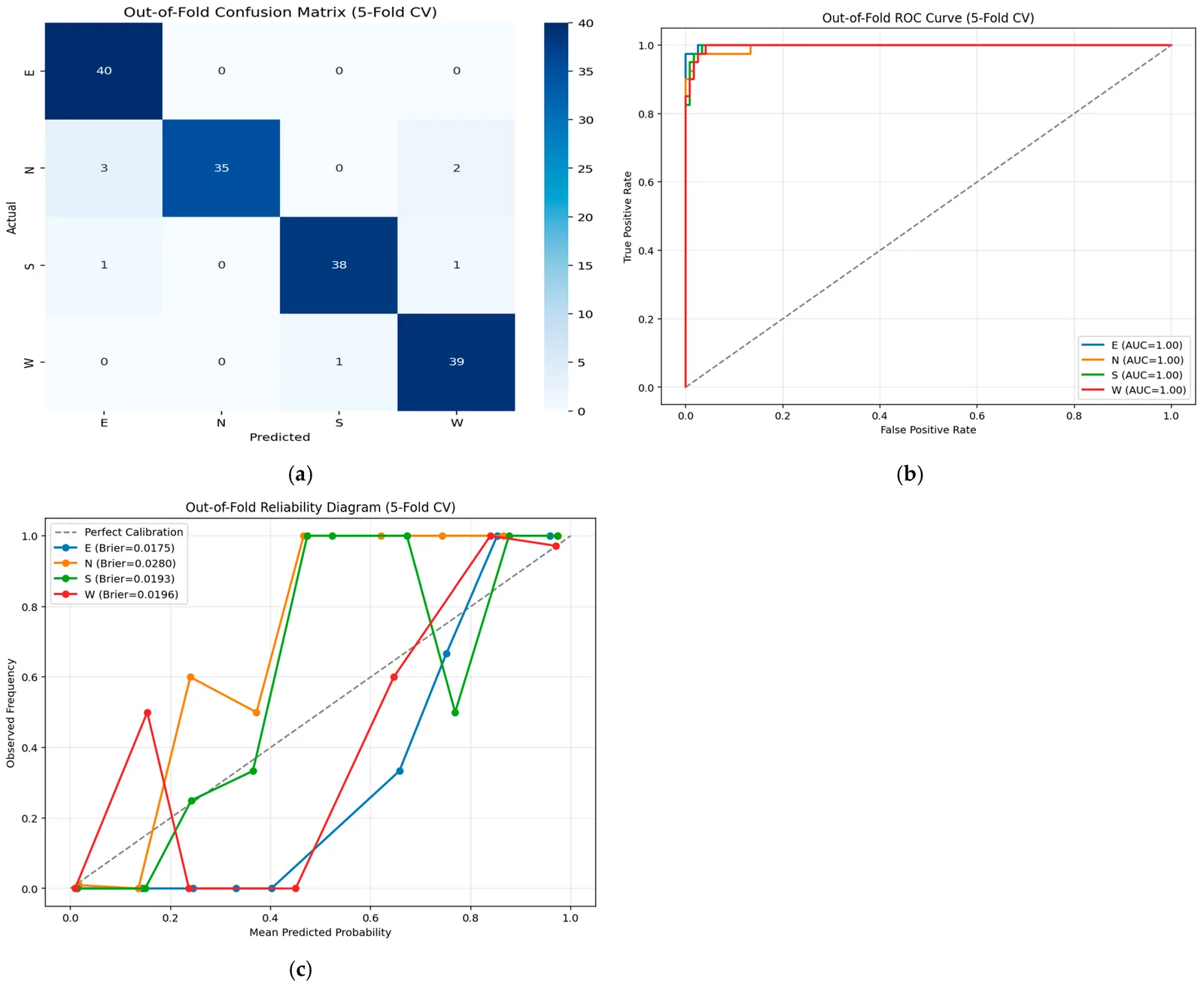
Statistical diagnostic panel for the proposed LightGBM framework for Aspect classification based on aggregated out-of-fold predictions generated through Stratified 5-Fold Cross-Validation: (**a**) confusion matrix displaying classification counts across four classes (E, N, S, W); (**b**) multi-class Receiver Operating Characteristic (ROC) curves with class-wise Area Under the Curve (AUC) values, where the dotted diagonal line represents the no-discrimination (random classifier) reference; and (**c**) reliability diagram and class-wise Brier scores assessing probability calibration.

To assess the class-specific performance and internal discriminative behavior of the proposed LightGBM framework for a different target attribute, a second experimental setup was evaluated for Aspect classification (Classes: E, N, S, W). Diagnostic performance metrics based on the aggregated out-of-fold predictions generated through Stratified 5-Fold Cross-Validation are presented in Figure 5.

- Confusion Matrix Analysis (Figure 5a): The out-of-fold confusion matrix obtained through Stratified 5-Fold Cross-Validation demonstrates strong classification performance across all four directional classes. Out of 160 observations, 152 were correctly classified, yielding an overall out-of-fold accuracy of 95.00%. The model correctly classified all 40 East (E) observations. For the North (N) aspect, 35 of 40 observations were correctly classified, with 3 misclassified as East and 2 as West. For the South (S) aspect, 38 of 40 observations were correctly classified, with 1 misclassified as East and 1 as West. For the West (W) aspect, 39 of 40 observations were correctly classified, with 1 misclassified as South. These results indicate consistently strong class discrimination within the present cross-validation framework.
- Multi-Class ROC Analysis (Figure 5b): Multi-class ROC analysis based on the aggregated out-of-fold probability estimates was performed to evaluate class-wise discrimination across the four spatial directions. The ROC analysis yielded AUC values that rounded to 1.00 for all four classes (E, N, S, and W), indicating very strong class-wise discrimination within the present out-of-fold evaluation.
- Model Calibration Assessment (Figure 5c): The out-of-fold reliability curves and class-wise Brier scores were used to assess the probabilistic performance of the model. The Brier scores were 0.0175 for East (E), 0.0280 for North (N), 0.0193 for South (S), and 0.0196 for West (W). These relatively low Brier scores indicate low overall probability prediction error within the out-of-fold evaluation. However, the class-specific reliability curves exhibit deviations from the ideal calibration line across some probability intervals; therefore, these findings should not be interpreted as evidence of perfect probability calibration.

## 4. Discussion

This study addresses the “black box” nature of machine learning in integrated pest management by identifying descriptive associations between observed Cherry Fruit Fly (*Rhagoletis cerasi* L.) pupal abundance and below-canopy soil conditions. The framework jointly profiles CfPC-defined density categories and directional bio-edaphic patterns using measured soil variables and interpretable IF–THEN rules.

Experiment 1 should be interpreted within its descriptive analytical design. Because continuous CfPC was used to define the pupal-density categories and was retained among the input variables, its dominant feature importance and the resulting high classification performance were expected. The analysis does not demonstrate that soil variables alone can predict unknown pupal density.

The corresponding pH, EC, lime, temperature, soil depth, and saturation ranges represent soil conditions that co-occurred with the observed CfPC-defined density profiles. These relationships should be regarded as site-specific statistical associations rather than independent causal or predictive thresholds.

Pupal viability, adult emergence, diapause development, soil oxygen concentration, puparial integrity, and fungal infection were not measured directly. Therefore, the relationships between soil properties and pupal abundance were interpreted as statistical field associations rather than experimentally demonstrated physiological mechanisms.

Experiment 2 was interpreted as an exploratory analysis of directional bio-edaphic differentiation rather than as an operational model for predicting an already known canopy direction. Its performance suggests that directional differences beneath the tree canopy were associated with distinguishable patterns in the measured biological and soil variables within the present dataset.

The agreement between the original orchard-level pupal-density pattern and the supplementary adult captures observed in 2026 (see Supplementary Materials) supports the potential biological relevance of soil pupal surveys. However, the adults captured in 2026 could not be attributed directly to the pupae recorded two years earlier. Extended diapause, pupal mortality, interannual climatic variation, host availability, and adult movement from surrounding orchards may have influenced the observed adult population. The 2026 observation was therefore interpreted as supportive evidence rather than independent temporal validation.

Ultimately, this XAI framework provides an interpretable approach for characterizing the bio-edaphic profiles associated with observed pupal-density levels. The extracted rules describe combinations of CfPC and soil conditions associated with these profiles and should not be interpreted as a prospective tool for identifying unknown high-risk pupal foci from soil properties alone. These interpretable patterns may provide a basis for the future development of localized precision crop-protection strategies following independent validation. Furthermore, the proposed XAI engine exhibits strong methodological universality: because the recursive path-traversal algorithm operates directly on hierarchical tree matrices (JSON), its application can be seamlessly adapted to other ensemble models such as XGBoost [31], CatBoost, and Random Forests, as well as broader tabular agricultural datasets.

Despite the strong internal classification performance and the interpretable bio-edaphic patterns identified by the rule-extraction framework, certain practical limitations must be acknowledged regarding model transferability. The rule-derived boundary values observed in this study reflect the specific bio-edaphic conditions of the studied local orchard matrix (e.g., specific soil texture, local microclimate, and cherry cultivar management). In real-world orchard systems, variations in clay-sand ratios, macro-climatic shifts, regional rainfall patterns, and rootstock differences may alter the exact numerical cut-offs of parameters such as saturation percentage or temperature. Nevertheless, while the specific quantitative thresholds may shift across different geographical regions, the observed associations between soil conditions and pupal abundance may reflect underlying bio-edaphic processes; however, the physiological mechanisms responsible for these patterns were not directly evaluated in this study and therefore require further experimental validation. Future studies should focus on validating and retraining this XAI framework across diverse soil taxonomies, varying climatic zones, and broader geographic regions to enhance its regional transferability and global IPM applicability.

## 5. Conclusions

This study presents an XAI framework that bridges the gap between complex machine learning algorithms and practical agricultural applications. By extracting highly reliable, human-readable IF-THEN decision rules from a high-performance LightGBM model using edaphic and biological predictors, the proposed approach enables the detailed analysis of Cherry Fruit Fly (Rhagoletis) habitats and can be readily extended to other precision agriculture management tasks. Within this unified pipeline, Experiment 1 characterized the bio-edaphic profiles associated with observed Low, Medium, and High pupal-density levels (CfPC), whereas Experiment 2 evaluated the classification of microclimatic aspects (Aspect) of cherry orchards.

When examining the primary contributions of the research, the transparency of the internal decision-making mechanisms of gradient-boosting decision trees, often described as “black boxes,” stands out. The developed rule-making engine allows agricultural experts and producers to directly interpret model decisions by converting complex mathematical matrices into clear physical threshold values. Experimental results have shown that edaphic variables such as soil temperature (T), pH, lime content (Lime), and saturation percentage (SY) were strongly associated with the observed pupal microhabitat patterns within the present dataset. Furthermore, the proposed LightGBM-based rule framework demonstrates high internal classification performance across both analytical scenarios, while the applied 5-fold cross-validation procedure provides an internal assessment of model stability and helps reduce the risk of overfitting. However, these results should not be interpreted as evidence of generalizability to independent external datasets, which requires further validation. These synthesized rules provide an interpretable representation of the bio-edaphic patterns observed beneath the tree canopy and may serve as a basis for the future development of localized decision-support strategies. However, independent validation across additional orchards, seasons, and environmental conditions is required before these rules can be used for operational identification of high-risk areas or targeted soil interventions.

While the study demonstrates high accuracy and explanatory power, it also has some limitations and areas for improvement that lay the groundwork for future research. Due to the dataset being limited to a specific geographic region and season, future studies will validate these derived rules across different climate zones and multiple seasonal intervals to improve spatio-temporal scalability. Furthermore, the developed rule engine is planned to be integrated with IoT-based real-time soil sensor networks; this will enable automated risk mapping of orchards and send instant pest density alerts to farmers’ mobile devices. Finally, the proposed flexible methodology aims to be extended to model the overwintering dynamics of other destructive orchard pests in the soil and to create a unified, explainable digital twin for precision crop protection processes. In conclusion, this study shows that predictive accuracy does not have to be sacrificed for the interpretability of the model, and thanks to the synergy provided by LightGBM’s explainable rule derivation, it offers an extremely robust, scientific, and practical paradigm for modern precision agriculture.

## Figures and Tables

**Table 1:** Descriptive statistics of the biological and soil physicochemical variables measured in 160 directional soil samples.

Feature	Unit	Avg.	Std. Dev.	Min.	Max.
CfPC	count (pupae)	3.32	1.63	0.00	10.00
Temperature	°C	11.19	0.61	10.20	12.70
pH	dimensionless	775.54	0.11	7.40	7.90
SoilDepth	m	0.31	0.19	0.10	0.70
EC	$\mathrm{dS}\;m^{-1}$	0.50	0.06	0.43	0.65
Lime	%	1.93	0.34	1.62	2.43
Saturation Percentage	%	42.69	2.77	38.10	49.00
**Feature**	**Avg.**	**Std. Dev.**		**Min.**	**Max.**
CfPC	3.32	1.63		0.00	10.00
Temperature	11.19	0.61		10.20	12.70
pH	7.54	0.11		7.40	7.90
SoilDepth	0.31	0.19		0.10	0.70
EC	0.50	0.06		0.43	0.65
Lime	1.93	0.34		1.62	2.43
SaturationPercentage	42.69	2.77		38.10	49.00
Dataset	Elazig-Harput Cherry Orchard Data				
Classes	Low/L, Medium/M, High/H				

**Table 2:** Hyperparameter settings and structural threshold configurations of the proposed LightGBM rule-extraction engine.

Component	Parameter	Variable	Value	Operational Purpose
Data Partitioning	Validation Split	test_size	0.20 (20%)	Allocates 80% for training and 20% for testing with stratified class balancing.
	Reproducibility Seed	random_state	42	Ensures deterministic splits and reproducible model weights.
LightGBM Ensemble	Base Classifier	LGBMClassifier	LightGBM	Handles non-linear edaphic and microclimatic interactions seamlessly.
	Number of Trees	n_estimators	50	Determines the total number of boosting iterations (trees to dump).
	Maximum Tree Depth	max_depth	5	Limits split levels to generate compact, interpretable human-readable rules.
	Step Size Shrinkage	learning_rate	0.1	Prevents overfitting by scaling the contribution of each new tree.
Rule Filtering Engine	Minimum Rule Accuracy	acc ≥ 75.0	75.0%	Eliminates weak logical pathways, selecting high-certainty boundaries.
	Minimum Sample Support	count ≥ 3	3 samples	Discards outlier/noisy nodes by ensuring the rule covers a stable sub-pattern.
	Duplicate Resolution	Unique Check	Strict Text Identity	Merges identical condition sets pulled from different boosting trees.
	Target Selection	Top Rules Limit	Array Slice [:10]	Isolates the absolute top 10 highest-accuracy operational rules.

**Table 3:** Highest-accuracy descriptive bio-edaphic rules selected after filtering and ranking, characterizing combinations of observed CfPC and soil conditions associated with the Low, Medium, and High pupal-density categories.

Rule	Class	Accuracy
IF 11.100 ≤ Temperature ≤ 12.700 AND 0.462 ≤ EC ≤ 0.464	L	1.00
IF 7.400 ≤ PH ≤ 7.550 AND 46.550 ≤ SaturationPercentage ≤ 49.000 AND 0.175 ≤ SoilDepth ≤ 0.700 AND 1.620 ≤ Lime ≤ 2.225 AND 0.432 ≤ EC ≤ 0.475	L	1.00
IF 7.400 ≤ PH ≤ 7.700 AND 38.400 ≤ SaturationPercentage ≤ 49.000 AND 0.432 ≤ EC ≤ 0.464 AND 10.200 ≤ Temperature ≤ 10.650	M	1.00
IF 11.150 ≤ Temperature ≤ 12.700 AND 43.250 ≤ SaturationPercentage ≤ 43.700	M	1.00
IF 10.200 ≤ Temperature ≤ 11.900 AND 38.100 ≤ SaturationPercentage ≤ 38.500 AND 0.565 ≤ EC ≤ 0.648	H	1.00
IF 7.450 ≤ PH ≤ 7.900 AND 12.050 ≤ Temperature ≤ 12.700 AND 38.100 ≤ SaturationPercentage ≤ 44.050 AND 0.100 ≤ SoilDepth ≤ 0.475 AND 1.820 ≤ Lime ≤ 2.430 AND 0.432 ≤ EC ≤ 0.462	H	1.00

Note: These rules represent descriptive profiles derived from the present dataset. Because the density classes were defined from continuous CfPC, the rules should not be interpreted as independent soil-based predictions of pupal density or as validated economic intervention thresholds.

**Table 4:** Class- specific classification performance metrics of the rule-based LightGBM model for Cherry Fruit Fly Pupal Concentration (CfPC) density class prediction scenario.

Pupal Density Class	Precision	Recall	F1-Score	Support
High (H)	1.0000	1.0000	1.0000	30
Low (L)	1.0000	1.0000	1.0000	50
Medium (M)	1.0000	1.0000	1.0000	80
Overall Accuracy	—	—	1.0000	160
Macro Average	1.0000	1.0000	1.0000	160
Weighted Average	1.0000	1.0000	1.0000	160

**Table 5:** Highest-accuracy agro-ecological decision rules selected after filtering and ranking from the LightGBM rule set for microclimatic Aspect (direction) prediction.

Rule	Class	Accuracy
IF 39.000 ≤ SaturationPercentage ≤ 49.000 AND 10.200 ≤ Temperature ≤ 11.650 AND 0.444 ≤ EC ≤ 0.458	S	1.00
IF 1.620 ≤ Lime ≤ 1.820 AND 10.200 ≤ Temperature ≤ 11.350 AND 7.400 ≤ PH ≤ 7.700 AND 0.468 ≤ EC ≤ 0.648 AND 0.100 ≤ SoilDepth ≤ 0.175 AND 44.700 ≤ SaturationPercentage ≤ 49.000	E	1.00
IF 41.600 ≤ SaturationPercentage ≤ 49.000 AND 0.000 ≤ CfPC ≤ 1.500 AND 11.650 ≤ Temperature ≤ 12.700	W	1.00
IF 1.820 ≤ Lime ≤ 2.430 AND 10.200 ≤ Temperature ≤ 10.850 AND 7.450 ≤ PH ≤ 7.900 AND 0.470 ≤ EC ≤ 0.648 AND 38.100 ≤ SaturationPercentage ≤ 41.650	N	1.00
IF 1.620 ≤ Lime ≤ 1.820 AND 0.470 ≤ EC ≤ 0.648 AND 0.475 ≤ SoilDepth ≤ 0.700 AND 41.750 ≤ SaturationPercentage ≤ 49.000 AND 0.000 ≤ CfPC ≤ 2.500	E	1.00
IF 38.100 ≤ SaturationPercentage ≤ 41.700 AND 7.550 ≤ PH ≤ 7.900 AND 10.200 ≤ Temperature ≤ 11.650	N	1.00
IF 10.950 ≤ Temperature ≤ 12.700 AND 1.820 ≤ Lime ≤ 2.430 AND 7.400 ≤ PH ≤ 7.450	W	1.00
IF 7.700 ≤ PH ≤ 7.900 AND 0.000 ≤ CfPC ≤ 3.500 AND 1.620 ≤ Lime ≤ 1.820 AND 0.470 ≤ EC ≤ 0.648	N	1.00
IF 0.461 ≤ EC ≤ 0.648 AND 11.000 ≤ Temperature ≤ 12.700 AND 7.400 ≤ PH ≤ 7.450	W	1.00
IF 1.820 ≤ Lime ≤ 2.430 AND 0.432 ≤ EC ≤ 0.462 AND 7.450 ≤ PH ≤ 7.900 AND 43.800 ≤ SaturationPercentage ≤ 49.000 AND 0.000 ≤ CfPC ≤ 4.500	S	1.00

**Table 6:** Class-specific classification performance metrics for the microclimatic Aspect prediction scenario.

Aspect Class	Precision	Recall	F1-Score	Support
East (E)	0.9091	1.0000	0.9524	40
North (N)	1.0000	0.8750	0.9333	40
South (S)	0.9744	0.9500	0.9620	40
West (W)	0.9286	0.9750	0.9512	40
Overall Accuracy	—	—	0.9500	160
Macro Average	0.9530	0.9500	0.9497	160
Weighted Average	0.9530	0.9500	0.9497	160

## Data Availability

The data presented in this study are available on request from the corresponding author. The data are not publicly available due to project and institutional restrictions.

## Supplementary Materials

The following supporting information can be downloaded at: https://www.mdpi.com/article/10.3390/insects17090946/s1.
